# Risperidone ISM Long-Acting as a Possible Treatment for Manic Episodes in Nonadherent Patients with Schizoaffective Disorder

**DOI:** 10.1192/j.eurpsy.2025.2063

**Published:** 2025-08-26

**Authors:** G. Barilla’, A. Cuomo, D. Koukouna, C. M. Borgese, A. Gallo, R. Larcinese, C. Venco, D. Bussolotti, A. Fagiolini

**Affiliations:** 1 U.O. Psichiatrica Mantova 1, ASST-Mantova, Mantova; 2 Molecular Medicine, University of Siena, Siena, Italy

## Abstract

**Introduction:**

Risperidone In Situ Microimplants (ISM®), a novel long-acting injectable (LAI) antipsychotic, rapidly achieves therapeutic plasma levels, with a significant first plasma peak occurring at 48 hours. This rapid onset of action, without the need for oral supplementation or loading doses, offers a promising new approach for effective management of acute symptoms (Walling et al. Drug Des Dev Ther 2021;15 4371-4382).

**Objectives:**

To evaluate the efficacy and safety of risperidone ISM (R-ISM) in the treatment of acute manic episodes with psychotic symptoms, focusing on symptom change over time.

**Methods:**

This preliminary and retrospective study included 15 inpatients with schizoaffective disorder who were started on R-ISM during a manic episode. RISM was administered after 6 days of oral risperidone. We retrospectively examined the Young Mania Rating Scale (YMRS) scores obtained in routine clinical practice at baseline (Dx), on the day of inpatient admission, on the day of injection (D0) and then 24 hours (D1), 48 hours (D2), 7 days (D8) and 28 days (D28) after the injection.

**Results:**

A statistically significant improvement in the total YMRS score was observed as early as the day of injection, with the median score decreasing from 37 [IQR: 4.5] at baseline to 27 [IQR: 2.5] at D0 and further to 20 [IQR: 4] at 24 hours after the injection 1 (D1) (p < 0.01). The improvement remained statistically significant at all assessment time points, reaching 11 [IQR: 1.5] at day 28 (D28) (*see Image 1*).

Single-item analysis showed rapid and significant improvement across all YMRS items, in particular the following symptoms (*see Image 2*):

- *Irritability:* Significantly decreased from a score of 6 [IQR: 0] at baseline to 3 [IQR: 0.5] at D1 and to 1 [IQR: 0.5] at D28 (p < 0.01).
- *Disruptive-Aggressive Behavior:* Significantly decreased from a score of 3 [IQR: 2] at baseline to 1 [IQR: 1] at D1 and to 0 [IQR: 1] at D28 (p < 0.01).
- *Sleep Disturbances:* Significantly decreased from a score of 3 [IQR: 1] at baseline to 0 [IQR: 0.5] at D28 (p < 0.01).
- *Speech (Rate and Amount):* Significantly decreased from a score of 4 [IQR: 1] at baseline to 1 [IQR: 0] at D28 (p < 0.01).
- *Content (Delusions; Hallucinations):* Significantly decreased from a score of 6 [IQR: 0.5] at baseline to 3 [IQR: 1] at D28 (p < 0.01).
- *Insight:* Significantly decreased from a score of 3 [IQR: 2] at baseline to 2 [IQR: 1] at D28 (p<0.01).

**Image 1:**

**Figure:**
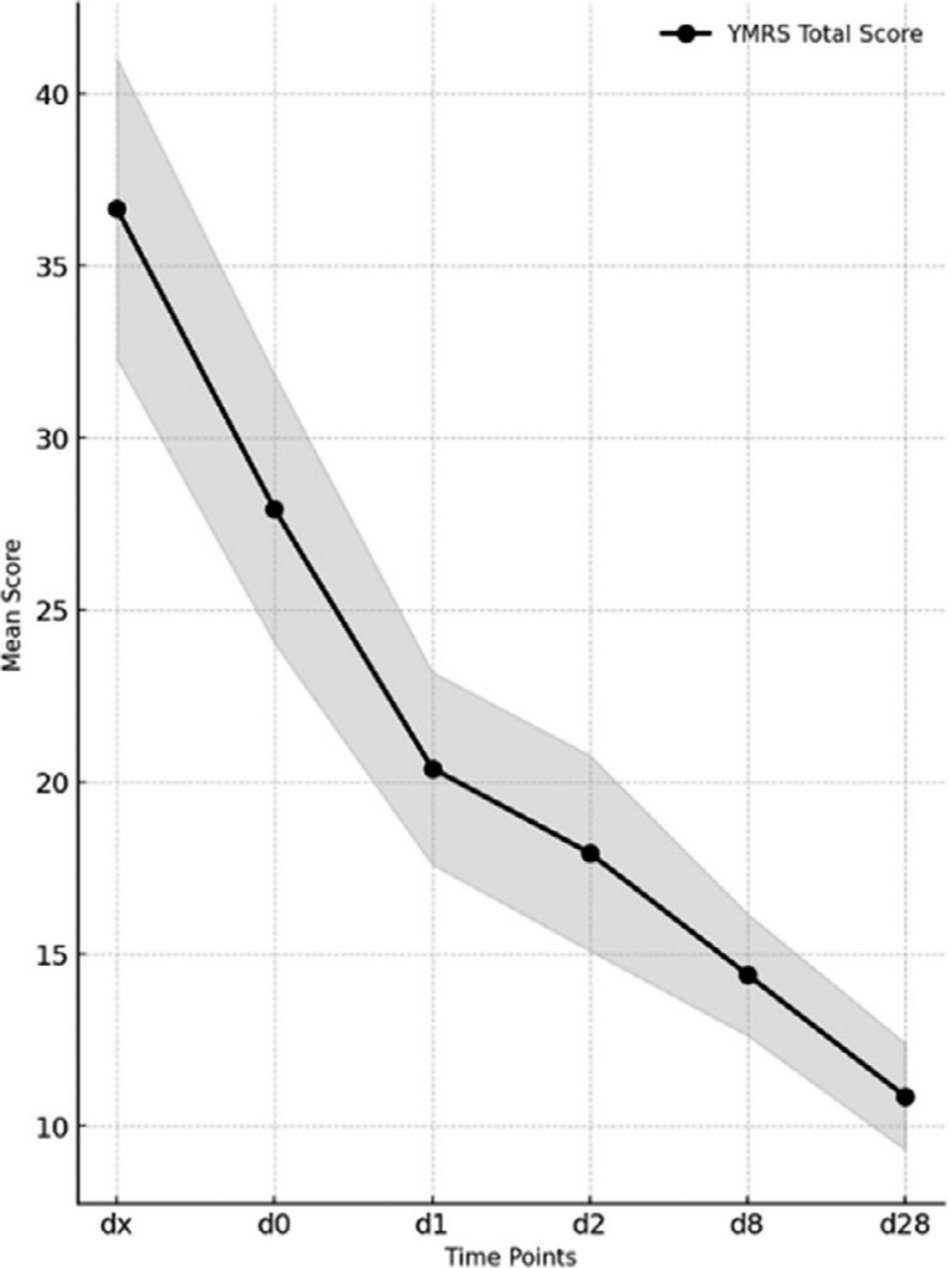

**Image 2:**

**Figure:**
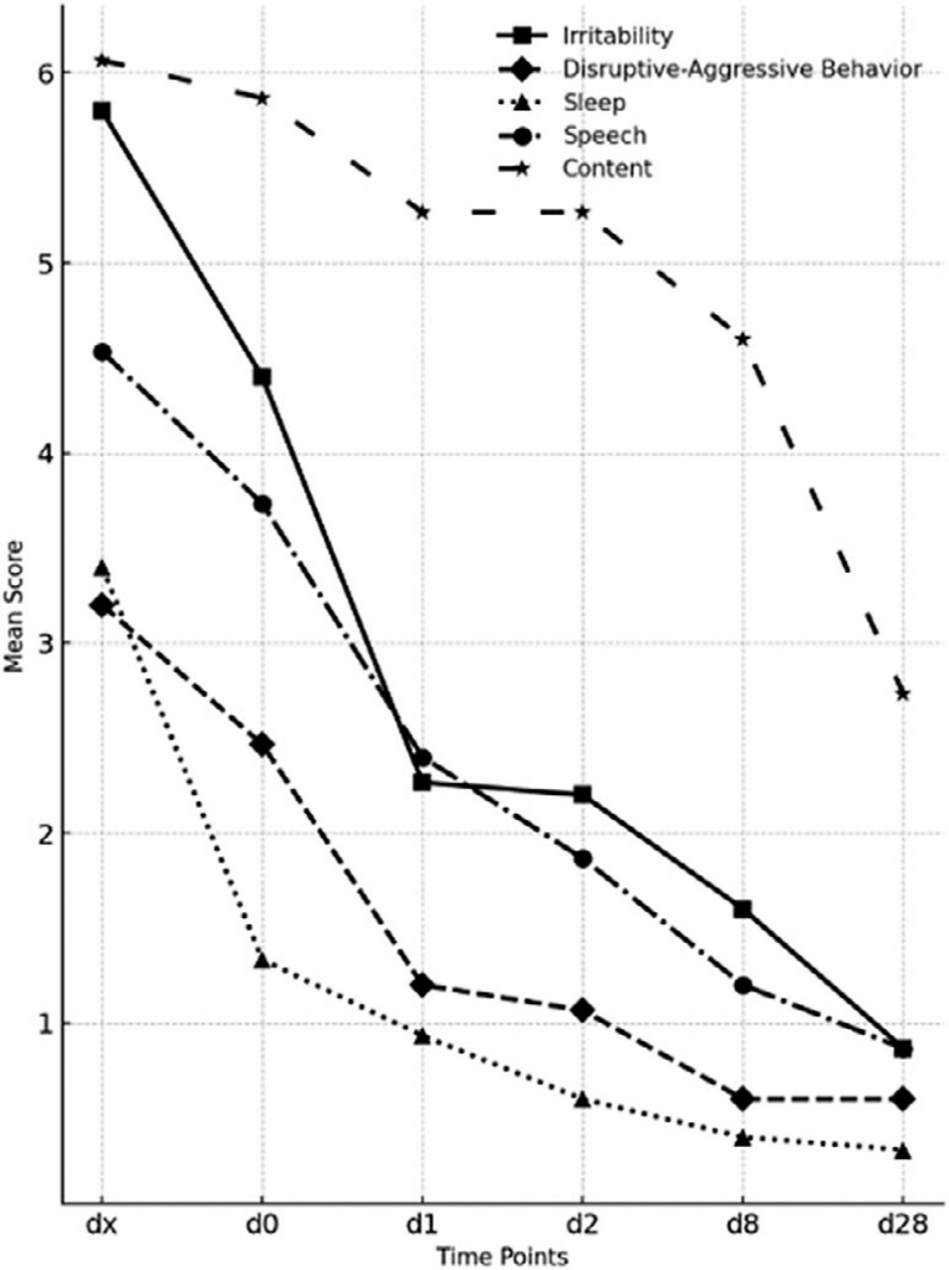

**Conclusions:**

This preliminary and retrospective study suggests the possible efficacy of risperidone ISM (approved for schizophrenia) for acute manic episodes. However, due to the retrospective design of the study, the small sample size, and the presence of concomitant treatments, the results are primarily exploratory and no conclusions can be drawn until prospective, randomized, placebo-controlled trials are conducted.

**Disclosure of Interest:**

None Declared

